# Correction of Ankle Malalignment in Severe Fibular Hemimelia

**DOI:** 10.1097/BPO.0000000000002876

**Published:** 2024-12-02

**Authors:** Milud Shadi, Piotr Janusz, Tomasz Kotwicki

**Affiliations:** Department of Spine Disorders and Pediatric Orthopedics, Poznan University of Medical Sciences, Poznan, Poland

**Keywords:** fibular hemimelia, FH, SUPERankle, congenital limb deficiency

## Abstract

**Background:**

Management of ankle joint deformity and instability are challenging issues in congenital fibular hemimelia (FH). This study aims to assess how much the SUPERankle procedure improves ankle alignment and provides durable ankle stability in patients with severe FH.

**Methods:**

Seventeen children aged 53.4±44.1 months with severe form of FH, equinovalgus foot deformation, ankle instability, and tibial curvature (Paley type IIIC), affecting 19 limbs, underwent the SUPERankle procedure. Foot and ankle position was evaluated clinically and radiologically before surgery, immediately after, and at follow-up of 63.0±19.7 months. Mechanical lateral distal tibial angle (mLDTA), tibiocalcaneal angle (mTCA), and tibiocalcaneal distance (mTCD) were measured on the AP radiograms, while the anterior distal tibial angle (mADTA) and lateral tibiocalcaneal angle (mLTCA) were measured on the lateral radiograms. Recurrences, additional procedures, and complications were documented based on medical records. Quality of life was evaluated with Limb Deformity-SRS questionnaire.

**Results:**

On clinical examination, the normal tibia and ankle alignment, along with a plantigrade foot were achieved in all limbs after the first surgery. In 11 limbs (58%) this result was maintained at follow-up. Due to recurrence, additional procedures were necessary to provide durable ankle alignment in 7 limbs (37%), while in 1 limb (5%) the ankle joint remained in equinus at the last follow-up. Significant improvement of radiologic alignment was found in all parameters (preoperative vs. postoperative vs. FU) as follows—mLDTA: 71.4±11.2 versus 88.7±5.6 versus 88.1±2.7 degrees, *P*=0.0001; mTCA: 41.4±14.9 versus 8.7±8.4 versus 11.6±8.9 degrees, *P*=0.0001; mTCD: 22.3±7.9 versus 4.0±3.6 versus 7.7±6.5 mm, *P*=0.0001; mADTA: 99.5±19.4 versus 82.3±4.2 versus 81.5±5.9 degrees, *P*=0.0002; mLTCA: 116.7±23.9 versus 95.8±11.7 versus 93.5±15.1 degrees, *P*=0.0002. The mean follow-up LD-SRS score was 4.03.

**Conclusion:**

In children with severe fibular hemimelia, the SUPERankle procedure provided clinically and radiologically fully corrected ankle joint and plantigrade foot, suitable for further lengthening procedure. The 40% rate of deformity recurrence was managed with additional surgical intervention to achieve a good clinical, radiologic, and functional outcome in 95% of children at 5-year follow-up.

**Level of Evidence:**

Level IV.

Fibular hemimelia (FH) is the most common type of congenital longitudinal lower limb deficiency, with a prevalence of 1:40,000 to 50,000 live births.^1,2^ FH can be a major part of the whole lower limb complex malformation rather than an isolated fibular deficiency. Severe FH which is classified by Paley type IIIC (Achterman and Kalamchi type II) is associated with lateral femoral condyle hypoplasia (valgus knee, knee instability), anteromedial bowing of the tibia, valgus and equinus of the hindfoot, ankle instability (both the clinical instability and the tendency for migration during the limb lengthening), tarsal coalition, lateral foot rays deficiency, and limb shortening.^3,4^ In addition, muscle malformation and fibrous remnant (anlage) predispose patients to recurrence of the deformity.^3^

Among several FH classification systems,^4–7^ the Achterman and Kalamchi^8^ is commonly used. More recently, Paley divided type III FH into 4 subtypes (A, B, C, D), each having different recommendation for surgical treatment.^3^ Paley type IIIC is defined by fixed equinovalgus deformity, with the deformity located in both the tibia and the hindfoot.^3^ Limb reconstruction of severe FH should address the entire complex deformity with the aim of restoring functional plantigrade foot, normal limb axis, and normal length. Soft tissue surgical procedures (posterolateral soft tissue release of the foot and ankle with the Achilles tendon and peroneal muscles tendons lengthening) followed by repeated lengthening procedures are typically used.^3,4^ One of the most challenging aspects of severe FH management is overcoming ankle joint instability and equinovalgus foot. The main obstacles comprise: tight lateral fibular anlage and its tethering effect, tight Achilles and peroneal muscles tendons, deficiency of hindfoot lateral support resulting from the lack of fibular malleolus, wedge shape of the distal tibial epiphysis, valgus foot deformity due to malorientation of the ankle and subtalar joints, and presence of a tarsal coalition along with missing lateral foot rays. Proper foot alignment should be achieved before undertaking leg lengthening. The SUPERankle preparatory surgical procedure is recommended at the age of 18 to 24 months.^3^ Failure to obtain primary plantigrade foot is the most common cause of unsatisfactory results in severe FH.

The purpose of this study was to assess how much the SUPERankle procedure improves ankle and foot alignment and provides durable ankle stability in children with severe FH (Paley type IIIC).

## METHODS

All consecutive patients presenting severe equinovalgus foot, ankle instability, and tibial anteromedial bowing in the frame of severe FH (Paley IIIC, Fig. 1) who were surgically treated at the department between September 2015 and September 2020 were included. All patients underwent the SUPERankle procedure consisting of: (1) total anlage excision, (2) tibial osteotomy for valgus and procurvatum correction and shortening, (3) osteotomy of the talocalcaneal coalition, (4) calcaneus positioning within the mechanical axis of the tibia, and (5) temporary intramedullary tibia fixation involving the hindfoot. The foot and ankle were transfixed with K-wires for 6 weeks and an above-the-knee cast was applied for 12 weeks. All surgeries were performed with 2 (medial and lateral) surgical approaches by the same experienced pediatric orthopaedic surgeon (the first author). Institutional Review Board approval was obtained for this study (No KB–635/23).

**FIGURE 1:**
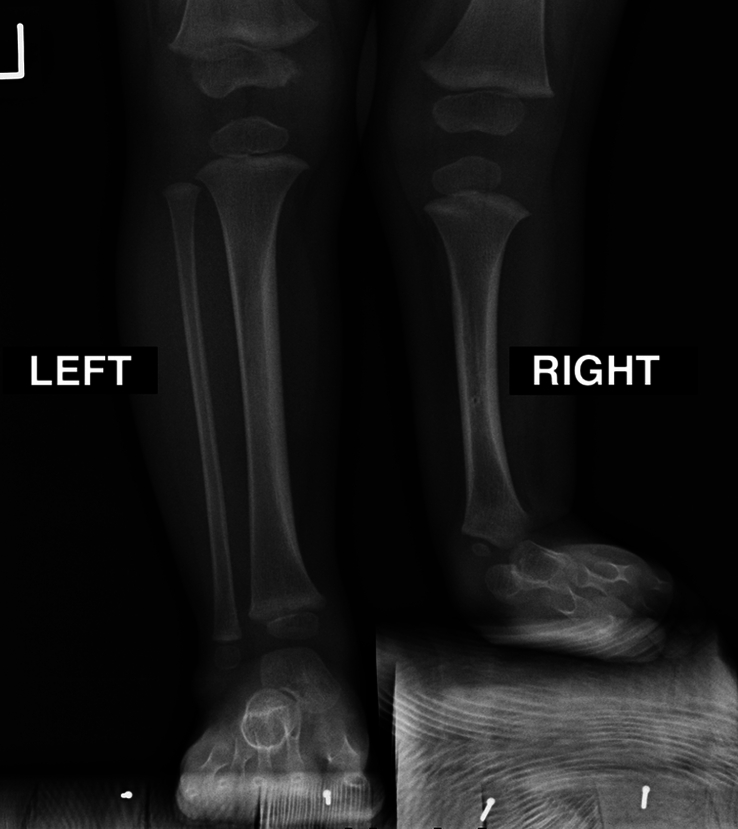
A twenty-two-month-old patient presenting severe fibular hemimelia (Paley IIIC type) of the right lower limb. A, Clinical image. B, Radiologic image (reverse orientation to match the clinical image).

### Clinical Evaluation

Clinical evaluation at follow-up included heel position assessment, equinus angle measurement, and heel valgus angle measurement in a standing position using a goniometer.

### Radiologic Evaluation

Preoperative and follow-up radiologic examination was performed with weight-bearing anteroposterior and lateral radiographs of the whole limb using a long cassette. Early postoperative radiologic assessment was performed in the plaster cast using nonweight-bearing radiographs.

After the SUPERankle procedure, 11 of 19 limbs underwent subsequent lengthening. Additional evaluation was performed twice in these patients: immediately before lengthening and 1 year after the external fixator removal.

The following radiologic measurements were selected based on literature and performed on the lateral radiograph (Fig. 2)^8,9^:

- Sagittal tibial angulation (sTA): the angulation of the tibial axis in the sagittal plane.
- Anatomic lateral tibiocalcaneal angle (aLTCA) measured between the distal cortical surface of the calcaneus and the anatomic axis of the distal diaphyseal segment of the tibia
- Mechanical lateral tibiocalcaneal angle (mLTCA) measured between the distal cortical surface of the calcaneus and the mechanical tibial axis.
- Anatomic anterior distal tibial angle (aADTA) formed by the anatomic axis of the distal diaphyseal segment of the tibia and the ankle joint orientation line in the sagittal plane.
- Mechanical anterior distal tibial angle (mADTA) formed by the mechanical axis of the tibia and the joint orientation line of the ankle in the sagittal plane.

**FIGURE 2:**
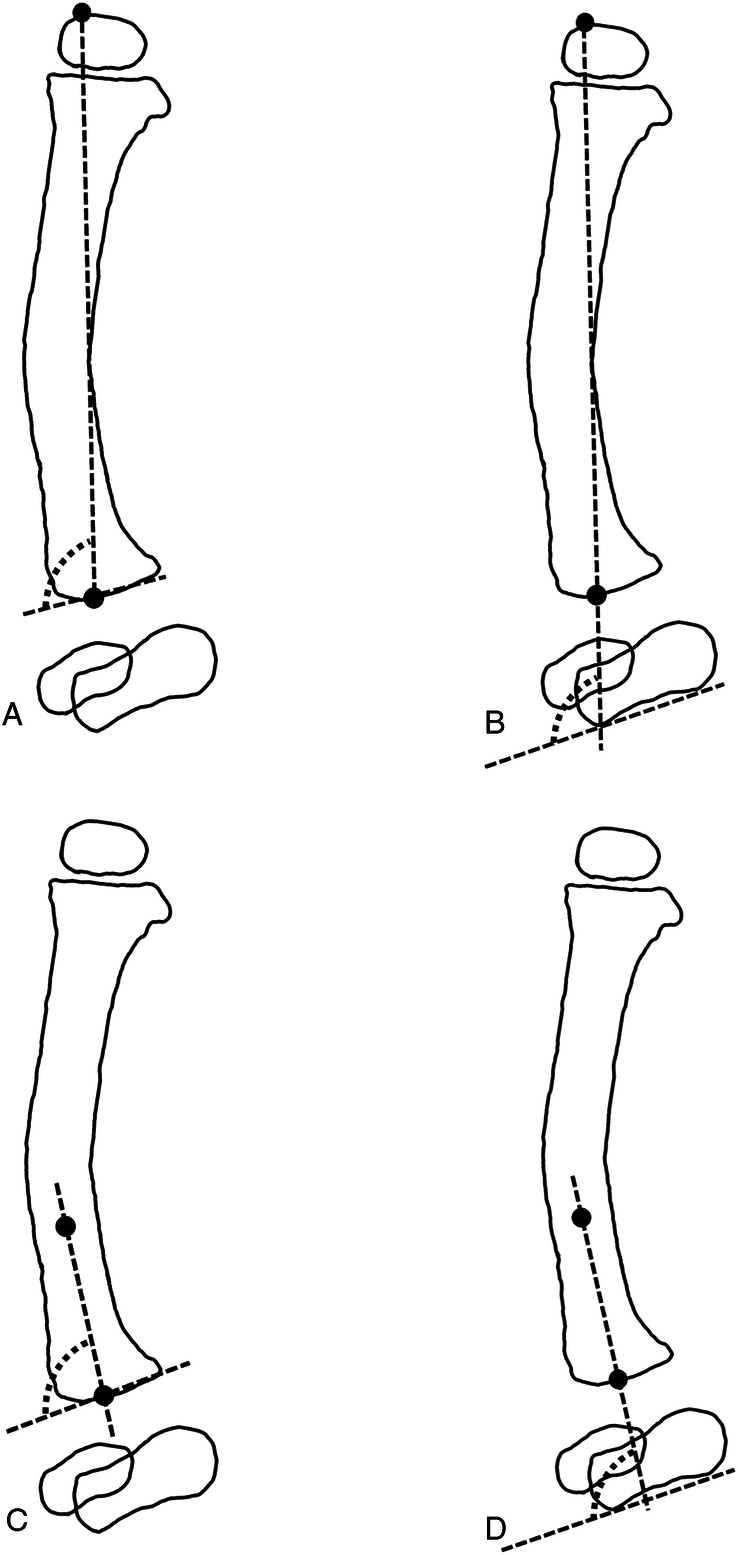
Radiologic measurement on lateral radiograph in sagittal plane. A, Mechanical anterior distal tibial angle (mADTA). B, Mechanical lateral tibiocalcaneal angle (mLTCA). C, Anatomic anterior distal tibial angle (aADTA). D, Anatomic lateral tibiocalcaneal angle (aLTCA).

The following radiologic measurements were selected based on literature and performed on the anteroposterior radiograph (Fig. 3)^8,9^:

- Tibial angulation (TA): the angulation of the tibial axis in the coronal plane.
- Anatomic lateral distal tibial angle (aLDTA) measured between the distal tibial articular surface and the anatomic axis of the distal diaphyseal segment of the tibia.
- Mechanical lateral distal tibial angle (mLDTA) measured between the distal tibial articular surface and the mechanical axis of the tibia.
- Anatomic tibiocalcaneal distance (aTCD) measured as the distance in mm between the anatomic axis of the distal diaphyseal segment of the tibia and the most distal point of the heel contacting the ground.
- Mechanical tibiocalcaneal distance (mTCD) measured as the distance in mm between the mechanical axis of the tibia and the most distal point of the heel contacting the ground.
- Anatomic tibiocalcaneal angle (aTCA) measured between the calcaneus anatomic axis and the anatomic axis of the distal diaphyseal segment of the tibia.
- Mechanical tibiocalcaneal angle (mTCA) measured between the calcaneus anatomic axis and the mechanical axis of the tibia.

**FIGURE 3:**
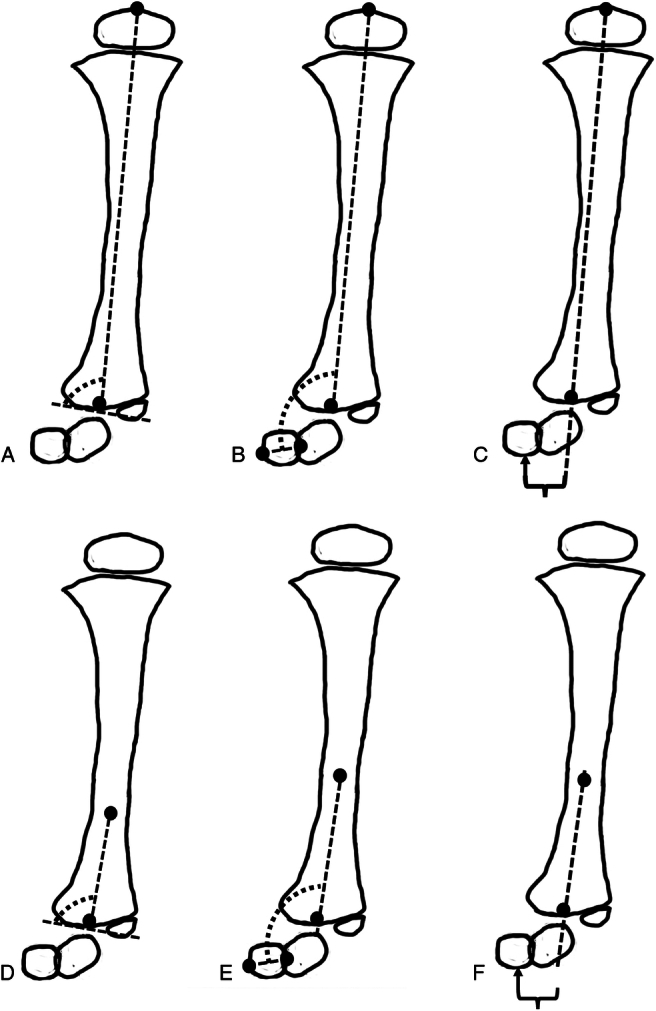
Radiologic measurement on AP radiograph in coronal plane. A, Mechanical lateral distal tibial angle (mLDTA). B, Mechanical tibiocalcaneal angle (mTCA). C, Mechanical tibiocalcaneal distance (mTCD). D, Anatomic lateral distal tibial angle (aLDTA). E, Anatomic tibiocalcaneal angle (aTCA). F, Anatomic tibiocalcaneal distance (aTCD).

The example of the radiologic measurements preformed in one of patients on radiographs before surgery and 1 year after surgery is shown in Figure 4. All radiologic measurements were performed by one orthopaedic surgeon with 15 years’ experience. The measurements were performed twice and the intraobserver interclass coefficient (ICC) was calculated (Supplemental Data 1, Supplemental Digital Content 1, http://links.lww.com/BPO/A830).^10^

**FIGURE 4:**
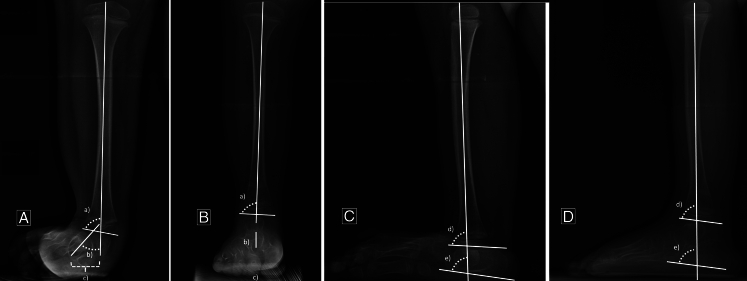
Radiologic measurement one of the patients before and 1 year after SUPERankle procedure. A, Radiologic measurement on AP radiograph in coronal plane before surgery—(a) mLDTA: 80 degrees; (b) mTCA: 40 degrees; (c) mTCD: 17 mm. B, Radiologic measurement on AP radiograph in coronal plane 1 year after surgery—(a) mLDTA: 89 degrees; (b) mTCA: 2 degrees; (c) mTCD: 0 mm. C, Radiologic measurement on lateral radiograph in sagittal plane before surgery—(a) mADTA: 85 degrees; (b) mLTCA: 80 degree. D, Radiologic measurement on lateral radiograph in sagittal plane 1 year after surgery—(a) mADTA: 82 degrees; (b) mLTCA: 82 degrees.

The recurrence was defined as a difference in radiologic measurements during follow-up >5 degrees.

It was not possible to perform radiologic measurements in 1 child (2 feet) due to lack of a visible calcaneus ossification center. This patient was excluded from the radiologic analysis.

### Quality of Life Evaluation

Patients’ quality of life was evaluated at follow-up visit using the Limb Deformity-Scoliosis Research Society (LD-SRS) questionnaire, which consists of 30 questions grouped in 5 domains (function, pain, self-image, mental health, and treatment satisfaction); higher points number signifying better quality of life.^11^

### Statistical Analysis

Mean values and SD were calculated. Kolmogorov-Smirnov test was used to assess for normal distribution. Paired samples *t* test or repeated analysis of variance (ANOVA) with Bonferroni correction were used to compare results having normal distribution. Wilcoxon or Friedman tests with Dunn post hoc test were used to compare nonnormal distribution data. Pearson correlation coefficient was used to compare preoperative and postoperative angles. Results were considered significant at *P*<0.05. Statistical analyses were performed using MedCalc 12.7.8.0 software.

## RESULTS

Twenty-three limbs in 20 children (4 girls and 16 boys) aged 48.7±41.4 months (range: 17 to 123 mo) at the time of surgery were operated on using the SUPERankle procedure. There were no infections, bone malunions or wound healing issues after the SUPERankle procedure. Two limbs were reoperated within 2 weeks due to destabilization of the tibial diaphyseal osteotomy and were restabilized with additional K-wires.

Three children dropped out: 2 children continued their treatment abroad (in USA) while in 1 child the parents declined further surgery in favor of using orthoprostesis. Thus, 19 limbs in 17 children (4 girls and 13 boys) aged 53.4±44.1 months were evaluated. The follow-up was 63.0±19.7 months (range: 24 to 85 mo).

The distribution of foot rays was as follows: 2 feet presented 2 rays, 14 presented 3 rays, 2 presented 4 rays, and 1 foot presented 5 rays. At follow-up, 18 limbs (95%) presented clinically plantigrade foot while 1 limb (5%) presented an equinus foot (Table 1).

**TABLE 1:** Patients’ Clinical Data

No	Affected limb/s	Operated limb	Gender	Number of foot rays	Age (mo)	Follow-up (mo)	Ankle position in coronal plane at the final follow-up/information of valgus recurrence during follow-up	Ankle position in sagittal plane at the final follow-up/information of equinus recurrence during follow-up	Lengthening	Problems and comments	Recurrence	Previous hindfoot and ankle surgery	LD-SRS
1	Both	R	M	3	123	83	RNS CO	RNS CO	N	Due to valgus limb axis treatment with 8-plate on tibia and femur during follow-up was performed	N	N	4.30
2	One	L	M	5	17	80	Recurrence of valgus CTA+10° after lengthening without clinical impact, CO	RNS CO	Y	Due to valgus limb axis treatment with 8-plate on tibia and femur during follow-up was performed Super hip super knee 6 years after SA	N	N	4.10
3	One	R	K	3	22	82	RNS CO	RNS CO	y	Due to valgus limb axis 8-plate on femur during follow-up was performed	N	N	4.07
4	One	R	M	3	22	71	RNS CO	RNS CO	Y	Due to valgus limb axis treatment with 8-plate on tibia and femur during follow-up was performed	N	N	3.90
5	One	R	M	3	115	82	Recurrence of the calcaneus valgus+14° CTA CO	Not fully corrected equinus 10° recurrence of CTA+10 after lengthening CO	Y	Not full correction of the equinus Deformation corrected after 2 years with distal tibia osteotomy. Further recurrence corrected with Ilizarov frame Due to valgus limb axis treatment with 8-plate on femur during follow-up was performed	Y	Y lengthening and correction with Ilizarov frame on tibia and foot	3.60
6	One	R	M	3	121	68	Recurrence+14° CTA CO	Recurrence+10° CTA after lengthening CO	Y	Recurrence after lengthening 3 years after SA, deformation corrected after 4 years with ankle arthrodesis Due to valgus limb axis treatment with 8-plate on femur during follow-up was performed Fracture after frame removal	Y	Y Foot surgery and tibia lengthening	4.40
7	One	L	M	2	121	71	CO	Residual equinus 18°, recurrence+11° CTA after lengthening CE 20°	Y	Due to valgus limb axis treatment with 8-plate on tibia and proximal tibia osteotomy during follow-up were performed	Y	Y Foot surgery and tibia lengthening	3.70
8	Both	L	K	3	87	47	RNS CO	RNS CO	N	Follow-up without additional procedures	N	N	3.93
9	Both	R	M	3	18	66	RNS CO	Residual equinus after surgery 12° and recurrence during follow-up+11° CO	N	Distal tibia deflexional osteotomy after 2 years Due to valgus limb axis treatment with 8-plate on tibia and femur during follow-up was performed	Y	N	4.27
10	Both	L	M	3	20	64	RNS CO	RNS CO	N	Follow-up without additional procedures concerning ankle. Forefoot correction	N	N	4.50
11	One	L	M	3	20	58	Recurrence of valgus before and after lengthening CTA+21° CO	Recurrence of equinus before and after lengthening+8°, CO	Y	Deformation corrected with second SA 3 years after primary surgery Due to valgus limb axis treatment with 8-plate on tibia during follow-up was performed	Y	N	3.67
12	One	R	M	3	21	26	Radiologic partial recurrence CTA+25° without clinical significance CO	RNS CO	Y	Due to valgus limb axis treatment with 8-plate on tibia during follow-up was performed	N	N	3.47
13	One	L	K	4	26	49	RNS CO	RNS CO	Y	Follow-up without additional procedures	N	N	3.70
14	One	R	M	2	25	48	Valgus recurrence after lengthening during follow-up CO	RNS CO	Y	Due to recurrence after lengthening 2 years after primary surgery, second SA procedure was performed Forefoot correction 4 years after SA Due to valgus limb axis treatment with 8-plate on femur during follow-up was performed	Y	N	4.20
15	One	R	K	5	62	65	RNS CO	RNS CO	N	Three years after SA SUPERhip and SUPERknee surgery was performed due to CFD and femur lengthening with MRS	N	N	3.70
16	Both	L	M	3	117	24	RNS CO	RNS CO	N	Follow-up without additional procedures	N	N	4.02
17	One	R	M	4	43	85	Valgus recurrence CTA+25° CO	RNS CO	Y	Deformation corrected with distal tibia osteotomy 3 years after SA, due to further recurrence ankle arthrodesis was preformed	Y	Y Foot surgery and tibia lengthening	4.20
18	Both	R	M	3	17	75	Recurrence of valgus 25° CO	Recurrence of equinus 30° CO	N	Very severe tibia shaft anteflexion (71°) and calcaneus equinus (clinically 90°) It was not possible to preform initial radiologic measurements due to lack of the visible calcaneus nucleus of ossification. Destablization of the tibia osteotomy implants due to very short bone segments. Requiring reoperation after 3 days with K wire Due to recurrence tibiocalcaneal arthrodesis was performed 2 years after SA Due to valgus limb axis treatment with 8-plate on tibia and femur during follow-up was performed	Y	N	4.30
19	Both	L	M	3	18	74	Residua valgus CTA 22° without clinical significance CO	CO	N	Patient with very severe tibia shaft anteflexion (68°) and calcaneus equinus (clinically 90°). It was not possible to preform initial radiologic measurements due to lack of the visible calcaneus nucleus of ossification Due to valgus limb axis treatment with 8-plate on tibia and femur during follow-up was performed	N	N	4.40

CE indicates clinical equinus position of the hindfoot; CO, clinical orthostatic position of the hindfoot; CV, clinical valgus position of the hindfoot; L, left; N, no; R, right; RNS, difference in radiologic measurements during follow-up <5 degrees; SA, SUPERankle; Y, yes.

Eleven patients (11 limbs) underwent subsequent lengthening using a spatial frame external fixator. In each case, the heel was fixed by the frame with 2 smooth K-wires in the neutral position. The heel fixation was removed 1 month after distraction was over. Leg lengthening of 59.1±14.3 mm (40.0 to 77.0 mm) was obtained. One tibial fracture occurred after frame removal. Thirteen patients underwent limb valgus correction using an 8-plate at the level of the medial distal femur.

In 11 limbs (58%) the clinical alignment of the tibia, ankle, and hindfoot was restored to correct position and maintained at final follow-up (mean: 5.2 y; range: 2.0 to 6.9 y) without additional surgery, even though 5 of the 11 limbs underwent leg lengthening.

In 8 limbs (42%) the deformity recurrence was noted. No difference was found in the age of patients with versus without recurrence at the time of surgery, mean 62.9 months (range: 17 to 121 mo) versus 48.8 months (range: 17 to 123 mo), respectively (*P*=0.93). Six of 11 limbs (54.5%) who underwent subsequent limb lengthening—presented deformity recurrence versus 2 of 8 patients (25%) presented recurrence without lengthening, *P*=0.35. We noticed deformity recurrence in 2 patients with a 2-ray foot (100%), in 5 patients with a 3-ray foot (35.7%), and 1 patient with a 4-ray foot (50%), Table 1. The recurrence deformity concerned 2 planes (valgus and equinus) in 4 feet, the valgus deformity in 2 feet, and the equinus deformity in 2 feet.

In 7 limbs out of 8 limbs with recurrence, the plantigrade foot position was obtained after 1 additional surgical procedure was performed while in 1 limb the foot equinus was still present at final follow-up. Secondary correction was achieved with the following additional procedures: a distal tibial osteotomy in 2 feet, a second SUPERankle procedure in 2 feet, and ankle arthrodesis in 3 feet (Table 1).

### Radiologic Findings

A substantial radiologic correction was identified in all limbs. The valgus axis of the ankle joint (aLDTA and mLDTA) and the tibial shaft curvature (TA) were corrected and the correction was maintained at follow-up (Table 2). The calcaneus valgus position was corrected and the calcaneus was centralized in line with the axis of the tibia, with mTCA reduced from 41.4 to 8.7 degrees and mTCD reduced from 22.3​​​​​​ to 4.0 mm (Table 2). The ankle joint position (ADTA) and tibial shaft curvature (sTA) were corrected and the correction was maintained in the sagittal plane at follow-up (Table 3). The equinus calcaneus position was significantly decreased and the improvement was maintained at follow-up.

**TABLE 2:** Coronal Plane Alignment

	Before SA (N=17)	Three days after SA (N=17)	Final follow-up (N=17)	*P*	*P* Comparison between before and after surgery	*P* Comparison between before surgery and final follow-up	*P* Comparison between after surgery and final follow-up
TA (deg.)	8.8±9.5 (0-28)	0.3±1.0 (0-4)	1.1±3.1 (0-12)	**0.0004** ^*^	**<0.001**	**<0.01**	>0.05
mLDTA (deg.)	71.4±11.2 (55-93)	88.7±5.6 (80-104)	88.1±2.7 (84-93)	**0.0001** ^†^	**<0.001**	**<0.001**	>0.05
mTCA (deg.)	41.4.±14.9 (13-65)	8.7±8.4 (0-23)	11.6±8.9 (3-35)	**0.0001** ^†^	**<0.001**	**<0.001**	>0.05
mTCD (mm)	22.3±7.9 (8-35)	4.0±3.6 (0-14)	7.7±6.5 (1-28)	**0.0001** ^†^	**<0.001**	**<0.001**	>0.05
aLDTA (deg.)	78.7±10.3 (60-93)	88.9±5.2 (82-104)	88.3±2.9 (84-94)	**0.0005** ^†^	**<0.01**	**<0.01**	>0.05
aTCA (deg.)	38.2±19.5 (7-66)	8.1±8.2 (0-23)	11.2±9.7 (0-35)	**0.0001** ^†^	**<0.001**	**<0.001**	>0.05
aTCD (mm)	19.5±6.7 (7-30)	3.8±3.2 (0-12)	6.6±3.9 (2-15)	**0.0001** ^†^	**<0.001**	**<0.001**	>0.05

* Friedman test with Dunn post-test *t*.

† Repeated ANOVA with Bonferroni correction, bold values *P*<0.05, SA-SUPERankle.

**TABLE 3:** Sagittal Plane Alignment

	Before surgery (N=17)	After surgery (N=17)	Final follow-up (N=17)	*P*	*P* Comparison between before and after surgery	*P* Comparison between before surgery and final follow-up	*P* Comparison between after surgery and final follow-up
sTA (deg.)	17.3±10.4 (0-33)	0.8±2.1 (0-6)	1.1±3.2 (0-12)	**0.0001** ^*^	**<0.001**	**<0.001**	>0.05
mADTA (deg.)	99.5±19.4 (73-144)	82.3±4.2 (73-88)	81.5±5.9 (71-93)	**0.0002** ^†^	**<0.01**	**<0.001**	>0.05
mLTCA (deg.)	116.7.±23.9 (78-164)	95.8±11.7 (75-117)	93.5±15.1 (79-133)	**0.0002** ^†^	**<0.01**	**<0.001**	>0.05
aADTA (deg.)	92.2±17.1 (73-133)	81.8±3.9 (73-88)	81.5±5.4 (70-89)	**0.0218** ^†^	>0.05	**<0.05**	>0.05
aLTCA (deg.)	107.3±20.0 (58-146)	95.5±11.3 (75-117)	93.4±14.0 (73-122)	**0.0083** ^†^	**<0.05**	**<0.05**	>0.05

* Friedman test with Dunn post-test.

† Repeated ANOVA with Bonferroni correction, bold values *P*<0.05.

Limb lengthening in 11 patients did not significantly impact radiologic measurements in the coronal plane (Table 4). Lengthening also did not impact tibial shaft curvature in the sagittal plane (sTA). A significant difference of 3.4 degrees was found in mADTA. The calcaneus equinus (mLTCA) increased nonsignificantly from 92.4 to 98.1, while the aLTCA increased significantly from 91.3 to 99.4 (Table 5).

**TABLE 4:** Coronal Plane Alignment Before and After Leg Lengthening

	Before elongation (N=12)	One year after elongation (N=12)	*P*
TA (deg.)	1.4.±3.1 (0-9)	0.7±1.3 (0-3)	0.75^*^
mLDTA (deg.)	85.4±4.7 (78-92)	85.7±3.8 (80-91)	0.89^†^
mTCA (deg.)	17.4±10.3 (0-28)	18.7±12.6 (4-42)	0.65^†^
mTCD (mm)	10.2±6.9 (2-20)	10.8±5.7 (2-20)	0.72^†^
aLDTA (deg.)	87.2±3.5 (82-92)	86.1±4.5 (80-94)	0.54^†^
aTCA (deg.)	16.2±10.8 (0-31)	17.4±13.3 (4-42)	0.67^†^
aTCD (mm)	9.8±6.9 (2-24)	10.2±5.6 (2-20)	0.79^†^

* Wilcoxon.

† Repeated *t* test.

**TABLE 5:** Sagittal Plane Alignment Before and After Leg Lengthening

	Before elongation (N=11)	One year after elongation (N=11)	*P*
sTA (deg.)	1.4±4.3 (0-13)	1.7±3.3 (0-8)	0.99^*^
mADTA (deg.)	81.0±7.6 (70-95)	84.4±5.2 (78-96)	**0.0367** ^†^
mLTCA (deg.)	92.4±16.2 (72-122)	98.1±19.9 (77-133)	0.27^†^
aADTA (deg.)	78.3±6.5 (70-92)	83.8±5.8 (78-96)	0.12^†^
aLTCA (deg.)	91.3±15.1 (72-122)	99.4±20.4 (76-133)	**0.0460** ^†^

* Wilcoxon.

† Repeated *t* test, bold values *P*<0.05.

### Quality of Life

The mean LD-SRS score was 4.03 (range: 3.47 to 4.50). Domain analysis showed that patients/parents were satisfied with the treatment. The mean satisfaction score was 4.40 (range: 3.33 to 5.0), and 94.7% of patients scored ≥4. The pain level score was 4.44 (range: 3.5 to 5.0), and 84.2% of patients scored ≥4. The function score was 4.05 (range: 2.86 to 4.57), 63.2%% of patients scored ≥4. Patients rated their mental health with a score of 3.89 (range: 2.40 to 5.00); 57.9% of patients scored ≥4. The lowest ratings were given to the self-image with a score of 3.69 (range: 3.11 to 4.33), 26.3% of patients scored ≥4.

The LD-SRS scores between patients without recurrence versus patients with recurrence did not differ significantly: LD-SRS 4.03 versus 4.03, *P*=0.97; function 4.02 versus 4.06, *P*=0.86; pain 4.54 versus 4.31, *P*=0.32; self-image 3.65 versus 3.74, *P*=0.48; mental health 3.87 versus 3.97, *P*=0.72; treatment satisfaction 4.33 versus 4.46, *P*=0.57. The LD-SRS scores were comparable between children without lengthening versus with lengthening: LD-SRS 4.08 versus 3.98, *P*=0.36; function 3.98 versus 4.06, *P*=0.72; pain 4.52 versus 4.39, *P*=0.20; self-image 3.72 versus 3.68, *P*=0.82; mental health 4.0 versus 3.82, *P*=0.16; treatment satisfaction 3.96 versus 4.33, *P*=0.48. No correlation of the LD-SRS scores versus age or ray number was observed.

## DISCUSSION

FH is a heterogenous congenital deformity with a wide spectrum of clinical presentation and disease course. In severe FH, the leg amputation stays a treatment option described in the literature. Amputation appears nowadays considered unacceptable in our society; parents asked for reconstructive treatment regardless the severity of the deformity. Two of our patients presented the foot with only 2 rays, which is generally considered to be nonfunctional.^5^ However, according to Fuller et al,^12^ if the foot is plantigrade it is usually functional regardless the ray number. According to Reyes et al^13^ it may be related with anatomic localization of the missing rays.

In our study, both patients with a 2-ray foot developed recurrence and both achieved plantigrade foot after the second surgery. According to Paley,^3^ the primary aim of FH management is a stable plantigrade foot, normal limb length and alignment, which should result in effective gait. We achieved this aim in 95% of our patients.

In this study, no knee joint subluxation nor knee joint stiffness following leg lengthening was observed, which was reported by Changulani et al^14^ as a significant problem.

Kulkarni et al^15^ described 10 patients classified as Paley type III who were treated with the SUPERankle procedure, 4 had recurrence of equinovalgus deformity (40%). We noted a similar recurrence rate of 42% (8 of 19 limbs). According to Kulkarni, ankle surgery before the age of 5 was less likely to result in recurrence.^15^ We found no age difference of patients with versus without recurrence, although patients without recurrence tended to be younger. We observed trend for more frequent recurrence in patients after lengthening, *P*-value not significant.

In this study, both anatomic and mechanical radiologic measurements were performed. In normal tibia the mechanical axis is parallel to the anatomic one; however, in severe FH there is tibial shaft angulation, such that anatomic measurements better express local ankle relations. After Popkov et al,^16^ we decided to consider a 5 degrees difference as significant. In 18 limbs only 1 tibial osteotomy was needed to obtain supramalleolar correction. In 1 case we had to perform double tibial osteotomy. The precise level and number of the osteotomy was based on preoperative on standing AP and lateral full limb length X-rays as well as on intraoperative ankle joint arthrography.

On clinical examination at the final follow-up 3 patients presented normal ankle joint and heel position however, the radiologic calcaneus valgus (CTA 20 to 25 degrees) was present. Similarly, in the sagittal plane, the calcaneus was observed to be more horizontal than in healthy patients (0 degrees inclination or lower); nevertheless, a clinically plantigrade foot was observed.

While the surgical procedure has been described previously,^3^ a few points of the technique are worth noting. The total anlage excision has been described as crucial technical point,^3^ and this was confirmed by Popkov et al.^16^ We agree with such approach, even if it was fused to the talocalcaneus complex and partial joint opening was needed. The procedure was primarily extra-articular, and we aimed to avoid interference with the ankle joint capsule. In cases of arthrotomy, the capsule was sutured or reconstructed with local soft tissues. The results of our tibial shaft axis and distal tibial configuration both postoperatively and at follow-up (aLDTA 88.9 and 88.3 degrees) were similar to those described by Popkov et al^16^ (88.5 and 87.1 degrees, respectively) in the group with anlage excision. Even after extensive anlage excision, we observed a trend toward progressive valgus knee deformity which is in line with data reported by Radler et al.^17^ Knee valgus was successfully managed by temporary hemiepiphysiodesis using an 8-plate. One of the clinical issues in FH is that the growth potential of the growth plates in the affected limbs is not only limited but also reveals asymmetry, resulting in a tendency for valgus deformity recurrence. Therefore, even after surgical treatment, there is a tendency for a rebound phenomenon throughout the growth period. This issue should be addressed as it appears during growth.

A tendency for hindfoot valgus during lengthening was observed, managed with hindfoot stabilization to the frame. Although the range of motion of the ankle joint was limited in all patients (there were no inversion and eversion motions, and dorsal and plantar flexion were close to 10 degrees), compensatory Chopart joint hypermobility was present. In our opinion, arthrodesis decreases foot function and should be avoided as long as other treatment options are possible. Indications for ankle arthrodesis include cases where the patient is unable to bear weight due to an unstable ankle or a severe foot deformity, and permanent immobilization of the ankle joint provides better function.

The procedure consisted of many steps among them lengthening of the peroneal muscles’ tendons. In many case 1 peroneal tendon was present, which was lengthened with the “Z” plasty. If there were 2 tendons, we lengthened it and combined into one.

According to the described procedure, the calcaneus should be positioned directly under the tibia in the coronal plane. Although perfect alignment is challenging, in our opinion, patients with postsurgical residual valgus >10 degrees are prone to recurrence. We aimed to achieve slight varus hypercorrection.

Taking into consideration the severity of the disease, the reported LD-SRS scores were surprisingly high. No control group was prepared which is a study limitation. According to Heath et al^18^ and Geenstein et al^19^ the mean LD-SRS score is 4.6 in heathy individuals. The LD-SRS score in our patients was similar to that described be Geffner et al^20^ after femoral derotation osteotomy. FH patients present reduced function compared with unaffected peers.^21^ Another study limitation is that QoL was assessed only at follow-up so potential changes cannot be evaluated. A limitation is the number of patients included which reflects the rarity of the disease; it represents the totality for our hospital since 2015. This group is comparable with Kulkarni et al^15^ when we consider the same disease severity. Another limitation of this study is the issue of the precision of the radiologic measurement in very young children. The shape of the ossified center may differ from the cartilaginous surface. However, most of the measurements revealed excellent (TA, mLDTA, mTCA, mTCD, aTCD, and sTA) and good repeatability (aLDTA, aTCA, mLTCA, and aLTCA) and only two (mADTA and aADTA) revealed moderate repeatability (Supplemental Data 1, Supplemental Digital Content 1, http://links.lww.com/BPO/A830).

## CONCLUSION

In children with severe fibular hemimelia, the SUPERankle procedure provided clinically and radiologically fully corrected ankle joint and plantigrade foot, suitable for further lengthening procedure. The 40% rate of deformity recurrence was managed with additional surgical intervention to achieve a good clinical, radiologic, and functional outcome in 95% of children at 5-year follow-up.

## Supplementary Material

SUPPLEMENTARY MATERIAL
