# Supplementary material for: Correction of Ankle Malalignment in Severe Fibular Hemimelia
Source: J Pediatr Orthop. 2024 Dec 2;45(4):e366–77. doi: 10.1097/BPO.0000000000002876 (PMC11875403; doi:10.1097/BPO.0000000000002876)
Supplement: SUPPLEMENTARY MATERIAL [file bpo-45-e366-s001.docx]

**Supplemental data**

Measurements were preformed twice by one researcher. Intraclass correlation coefficient was calculated for the measurements values. The results are presented in Table S.

Table S. Results of the intraobserver intraclass correlation coefficient for radiological measurements

|  | Intraclass correlation coefficient  N=34 | | 95% Confidence Interval |
| --- | --- | --- | --- |
| TA | 0.9129 | | 0.8209 to 0.9579 |
| mLDTA | 0.9273 | | 0.8489 to 0.9650 |
| mTCA | 0.9517 | | 0.9006 to 0.9766 |
| mTCD | 0.9558 | | 0.9091 to 0.9786 |
| aLDTA | 0.7871 | | 0.5566 to 0.8976 |
| aTCA | 0.8411 | | 0.6685 to 0.9236 |
| aTCD | 0.9729 | | 0.9433 to 0.9870 |
| sTA | | 0.9624 | 0.9199 to 0.9824 |
| mADTA | | 0.7091 | 0.3903 to 0.8604 |
| mLTCA | | 0.7997 | 0.5897 to 0.9029 |
| aADTA | | 0.7295 | 0.4389 to 0.8696 |
| aLTCA | | 0.7608 | 0.5031 to 0.8847 |

Abbreviations and description of measurements:

- sagittal tibial angulation (sTA) – the angulation of the tibial axis in the sagittal plane.
- anatomical lateral tibiocalcaneal angle (aLTCA) measured between the distal cortical surface of the calcaneus and the anatomical axis of the distal diaphyseal segment of the tibia
- mechanical lateral tibiocalcaneal angle (mLTCA) measured between the distal cortical surface of the calcaneus and the mechanical tibial axis;
- anatomical anterior distal tibial angle (aADTA) formed by the anatomical axis of the distal diaphyseal segment of the tibia and the ankle joint orientation line in the sagittal plane;
- mechanical anterior distal tibial angle (mADTA) formed by the mechanical axis of the tibia and the joint orientation line of the ankle in the sagittal plane;
- tibial angulation (TA) – the angulation of the tibial axis in the coronal plane.
- anatomical lateral distal tibial angle (aLDTA) measured between the distal tibial articular surface and the anatomical axis of the distal diaphyseal segment of the tibia;
- mechanical lateral distal tibial angle (mLDTA) measured between the distal tibial articular surface and the mechanical axis of the tibia;
- anatomical tibiocalcaneal distance (aTCD) measured as the distance in mm between the anatomical axis of the distal diaphyseal segment of the tibia and the most distal point of the heel contacting the ground;
- mechanical tibiocalcaneal distance (mTCD) measured as the distance in mm between the mechanical axis of the tibia and the most distal point of the heel contacting the ground;
- anatomical tibiocalcaneal angle (aTCA) measured between the calcaneus anatomical axis and the anatomical axis of the distal diaphyseal segment of the tibia;
- mechanical tibiocalcaneal angle (mTCA) measured between the calcaneus anatomical axis and the mechanical axis of the tibia.

Results interpretation:

- values less than 0.5 are indicative of poor reliability,
- values between 0.5 and 0.75 indicate moderate reliability,
- values between 0.75 and 0.9 indicate good reliability,
- values greater than 0.90 indicate excellent reliability^1^.

Supplement references

1. Koo TK, Li MY. A Guideline of Selecting and Reporting Intraclass Correlation Coefficients for Reliability Research. *Journal of Chiropractic Medicine* 2016;15:155–63.
